# Ebola Virus Persistence in Immune-Privileged Niches: Molecular Mechanisms, Host–Virus Interactions, and Therapeutic Opportunities

**DOI:** 10.3390/microorganisms14102312

**Published:** 2026-10-10

**Authors:** Awadh Alanazi, Wejdan Oudah Albalawi, Eid S. Alshammari, Maram Awied Alenezi, Hanaa Elsayed Ahmed Shahin, Mohamed N. Ibrahim

**Affiliations:** 1 Department of Clinical Laboratory Sciences, College of Applied Medical Sciences, Jouf University, Sakaka 72388, Saudi Arabia; 2 Department of Clinical Laboratory Sciences, College of Applied Medical Sciences at Al Qurayyat, Jouf University, Al Qurayyat 77454, Saudi Arabia; 3 Northern Borders Health Cluster, Almosaediah, Arar 73311, Saudi Arabia; 4 Nursing Department, College of Applied Medical Sciences at Al Qurayyat, Jouf University, Al Qurayyat 77454, Saudi Arabia; 5 Microbiology Department, Faculty of Science, Ain Shams University, Cairo 25611, Egypt

**Keywords:** Ebola virus, immune privilege, viral persistence, host–pathogen interaction, therapeutic opportunities

## Abstract

Ebola virus disease (EVD) has traditionally been conceptualized as an acute, fulminant illness culminating in either death or apparent systemic viral clearance. However, clinical, virological, and genomic evidence gathered since the 2013–2016 West African epidemic demonstrates that Ebola virus (EBOV) can persist for months to years within immunologically privileged anatomical compartments, particularly the central nervous system, eye, and male reproductive tract, after blood viremia becomes undetectable. Importantly, the strength of evidence differs by endpoint: RT-PCR positivity demonstrates persistence of viral RNA, recovery in culture demonstrates replication-competent virus, and documented transmission requires epidemiological and, where available, genomic linkage. Persistent infection has been associated with clinically important outcomes, including relapsing meningoencephalitis, genomically supported sexual transmission long after recovery, and outbreak resurgence linked to persistent human viral lineages. Synthesizing evidence across human cohort studies, case reports, non-human primate models, and organoid systems, this review examines the anatomical, molecular, and immunological factors that may permit EBOV survival in privileged niches. Structural barriers and local immunoregulatory environments are established features of these compartments, whereas the contributions of defective viral genomes, cell-type-specific interferon responses, exosome- or cell-mediated spread, host immunogenetic variation, and acute-phase treatment to long-term reservoir establishment remain supported to differing degrees and, in several cases, are still mechanistic hypotheses. Although monoclonal antibodies and direct-acting antivirals have transformed acute-phase outcomes, their effects on established tissue reservoirs remain incompletely defined. key knowledge gaps establishing the relationship between viral RNA detection and true infectivity, defining correlates of protection against relapse, and determining how acute-phase therapeutic choice influences reservoir establishment remains essential for developing compartment-penetrant treatments, updating survivor care guidelines, and preventing future outbreak resurgences.

## 1. Introduction

Ebola virus was first identified in 1976 during simultaneous outbreaks in Yambuku, Zaire (now the Democratic Republic of the Congo, DRC), and Nzara, South Sudan. The Yambuku outbreak, amplified by unsterilized needle reuse in a rural mission hospital, caused over 300 infections with high mortality; investigators isolated a novel filamentous virus, morphologically similar to Marburg virus, and named it after the nearby Ebola River [1]. EBOV (historically referred to as Zaire ebolavirus) has caused several of the largest recorded EVD outbreaks, and historical case-fatality rates have varied substantially across outbreaks and settings [2].

From 1976 to 2013, Ebola emerged as sporadic, geographically limited outbreaks of disease in central Africa, including the Kikwit outbreak of 1995 (315 cases, 250 deaths) that was contained through isolation, contact tracing, and safer burial practices. The 2013–2016 West African epidemic was the largest recorded EVD epidemic, with more than 28,600 cases and 11,300 deaths, occurring in the context of high population mobility, porous borders, weak health systems, and delayed diagnosis [3]. Importantly, the large number of survivors enabled longitudinal studies of post-Ebola outcomes at an unprecedented scale [4]. Persistent infection subsequently became epidemiologically important because genomically supported survivor-associated transmission and outbreak resurgence were documented after apparent recovery [5,6].

Acute EVD occurs after a 2–21-day latent period, and features fever, weakness, myalgia, and gastrointestinal symptoms, often resulting in multiple organ failure, coagulopathy, shock and mortality within 6–16 days; modern treatment relies on viral clearance, adaptive immunology, supportive therapy and monoclonal antibody therapy [7,8]. Recovery in clinic, however, is seldom back to normal. The survivors experience Post-Ebola Syndrome (PES) between 50% and 75% of the time, which includes chronic arthralgia, myalgia, headache, sensorineural hearing loss, sleep disruption and neuropsychiatric disturbance [9]. Eye problems occur in 13–34% of survivors, with about 40% of those with eye problems having vision loss. Although there is general improvement in constitutional symptoms over time, ocular and neurological symptoms do not change course and characterize two distinct etiologic groups in PES: post-infectious inflammatory sequelae on one hand, and manifestations of persistent, low-grade viral replication in tissue-sequestered, immune-privileged niches on the other [10]. This review examines current evidence of the existence of such niches, the molecular mechanisms and host–virus interactions that allow viruses to persist for extended periods of time, and the major gaps in knowledge and new therapeutic strategies that will be important to address in the elimination of persistent reservoirs, the prevention of viral relapse, and the reduction in transmission by survivors.

Immune privilege refers to anatomically and physiologically unique tissue compartments, the barriers and distinct lymphatic drainage of which, in addition to limited expression of MHC class I/II, active immunosuppressive mechanisms act to limit or provide some deviation from conventional immune surveillance, most notably within the central nervous system, eye, testis and fetoplacental unit [11]. The molecular and cellular features pertinent to this review are: physical barriers that block the passage of immune cells, antibodies and complement within the tissue (blood–brain, blood–CSF, blood–ocular, and blood–testis barriers); constitutive expression of low levels of MHC class I and II on the parenchymal cells, which limits the recognition by the cytotoxic T lymphocytes; local expression of Fas ligand (FasL) that can induce the apoptosis of infiltrating Fas-bearing lymphocytes; secretion of immunosuppressive mediators such as TGF-β and α-melanocyte-stimulating hormone (α-MSH); and anterior chamber-associated immune deviation (ACAID), which actively induces a systemic response deviated away from delayed-type hypersensitivity and towards tolerance, in the eye specifically [12].

## 2. Genomic Evidence Establishing EBOV Persistence

Evidence for EBOV persistence should be interpreted according to the virological endpoint measured. Detection of EBOV RNA by RT-PCR demonstrates the presence of viral genetic material but does not, by itself, establish the presence of replication-competent virus or infectiousness. Recovery of viable virus by culture provides stronger evidence of ongoing biologically active infection, whereas demonstrated transmission additionally requires epidemiological and, where available, genomic evidence linking donor and recipient infections. For interpretive clarity, the evidence discussed in this review is considered hierarchically: (i) clinical observations and RT-PCR detection; (ii) direct recovery of replication-competent virus; (iii) genomic evidence linking persistent lineages to relapse or transmission; (iv) longitudinal human and experimental reservoir studies; and (v) mechanistic hypotheses derived from observational, animal, organoid, or cross-viral evidence. Mechanistic proposals are not considered equivalent to demonstrated human causal mechanisms. Direct evidence of ocular persistence was established by Varkey et al., who recovered viable EBOV from the aqueous humor of a convalescent patient with severe panuveitis at a time when blood RT-PCR was negative [13]. CNS persistence was documented in a recovered patient who developed late meningoencephalitis approximately nine months after recovery, with high EBOV RNA levels in cerebrospinal fluid and whole-genome sequencing confirming direct descent from the original infecting strain [14]. Longitudinal semen studies in Sierra Leone, Liberia, and Guinea demonstrated prolonged EBOV RNA detectability, while selected transmission events were supported by epidemiological and genomic evidence, including transmission linked to a survivor more than 500 days after illness onset [5]. Most importantly, whole genome sequencing of the 2021 outbreaks in Guinea and the DRC showed viral lineages with sub-expected divergence from their 2013–2016 and 2018–2020 origins, respectively, suggesting that epidemics might be reactivated from human reservoirs rather than new zoonoses a paradigm-shifting shift in outbreak surveillance [6].

## 3. Molecular Virology and Pathobiology

**Figure 1:**
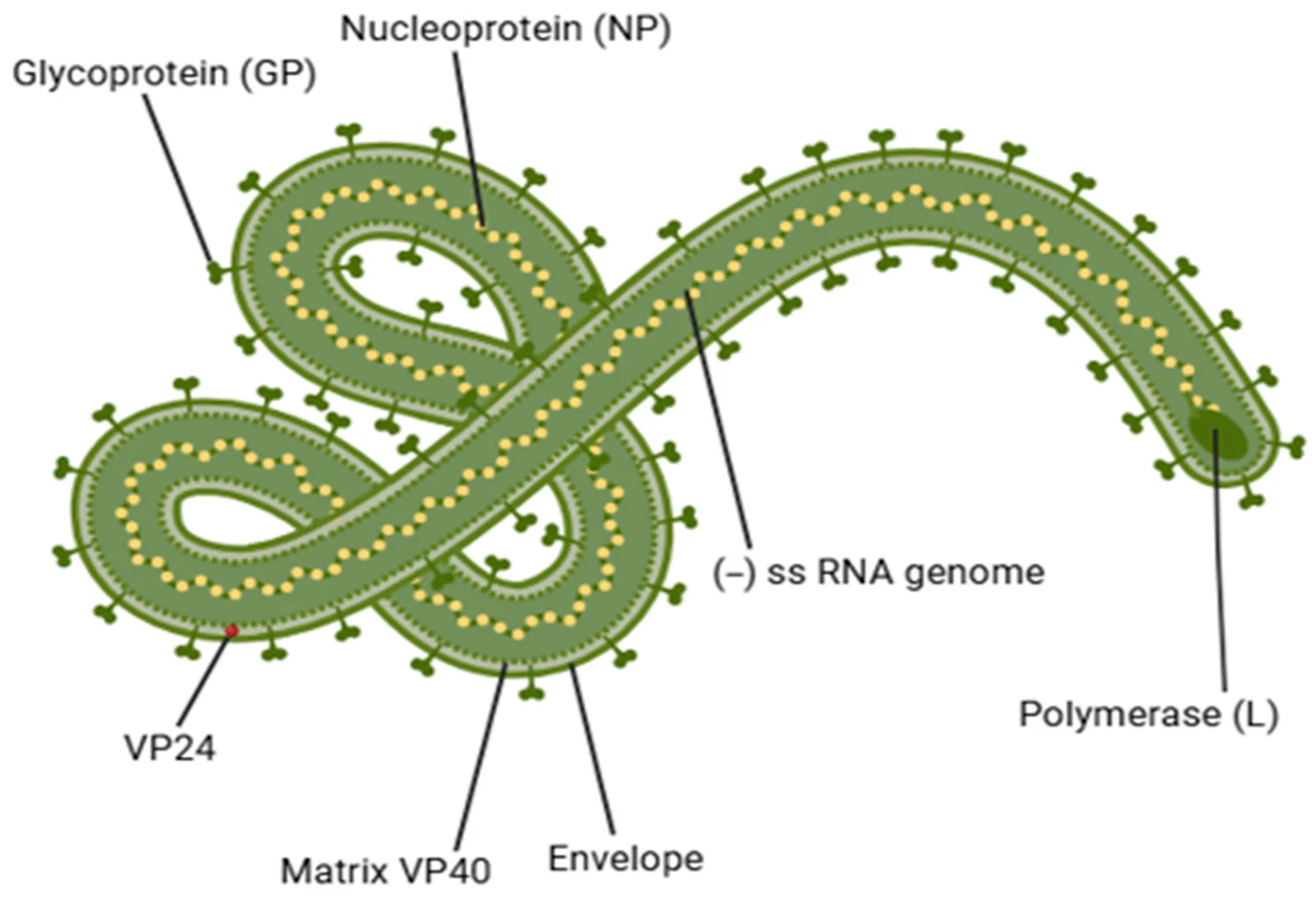
Structure of Ebola Virus. Created in BioRender. Ibrahim, M. (2026). https://BioRender.com/oykfpz3.

EBOV is a negative-sense, single-stranded RNA virus of the family Filoviridae with a genome of approximately 19 kb encoding seven principal structural proteins: nucleoprotein (NP), VP35, VP40, glycoprotein (GP), VP30, VP24, and the RNA-dependent RNA polymerase (L) [15]. RNA editing of the *GP* gene generates additional glycoprotein products, including soluble GP (sGP), which has immunomodulatory functions [16]. Viral transcription and genome replication occur in cytoplasmic inclusion bodies containing the nucleocapsid and the VP35–VP30–L polymerase complex; VP30 RNA binding is important for transcriptional activation [17,18]. VP24, in contrast, is best established as a structural and interferon-antagonist protein and should not be presented as a direct determinant of defective viral-genome formation [19,20]. The principal structural components and genomic organization of EBOV relevant to these processes are summarized in Figure 1.

**Figure 2:**
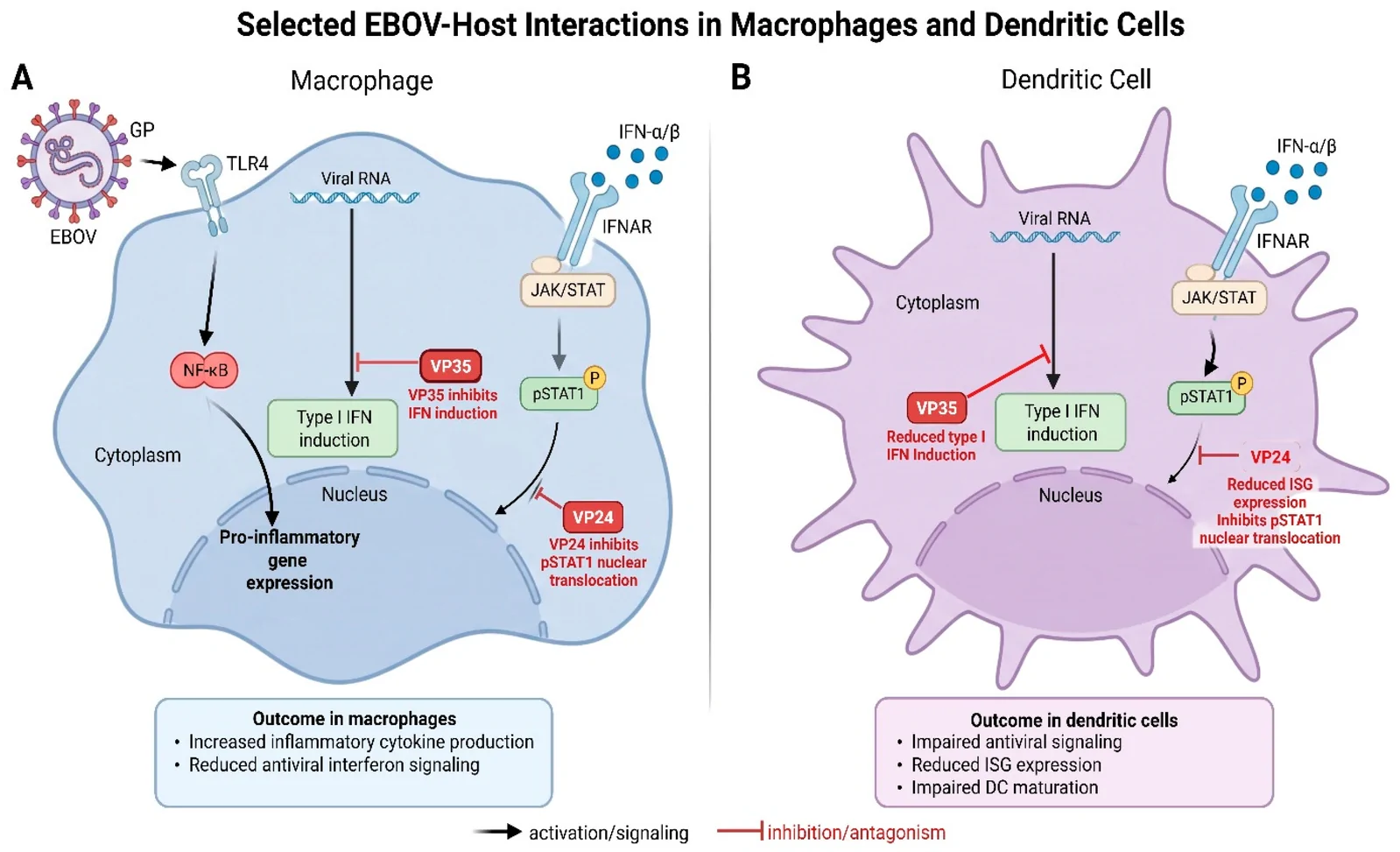
Selected EBOV–host interactions involved in inflammatory activation and interferon antagonism. (**A**) Selected viral–host interactions in macrophages; (**B**) selected viral–host interactions in dendritic cells. Created in BioRender. Ibrahim, M. (2026). https://BioRender.com/gv27zef.

EBOV entry is a multistep process. Initial cellular uptake can occur through a macropinocytosis-like pathway [21]. Following trafficking to acidified endosomal compartments, GP is proteolytically processed and engages Niemann–Pick C1 (NPC1), an essential intracellular receptor for filovirus entry [22]. Subsequent GP2-mediated membrane fusion releases the viral ribonucleoprotein complex into the cytoplasm. Because NPC1 is broadly expressed, its use is compatible with the wide cellular tropism of filoviruses, although receptor expression alone does not determine which cell types support long-term persistence [22]. EBOV modulates innate immune signaling through several distinct viral–host interactions. In macrophages, EBOV glycoprotein can promote TLR4-dependent inflammatory signaling, whereas VP35 and VP24 antagonize antiviral interferon pathways through different mechanisms. VP35 primarily suppresses upstream interferon induction, while VP24 inhibits nuclear translocation of phosphorylated STAT1. In dendritic cells, these viral antagonists contribute to impaired antiviral signaling and defective maturation. These selected, cell-context-dependent interactions are summarized in Figure 2. The schematic is based on published mechanistic studies of EBOV GP–TLR4 signaling and VP35/VP24-mediated interferon antagonism [19,23,24,25,26].

Genome replication is carried out by the L protein together with VP35 and VP30 [18]. Defective viral genomes (DVGs) can arise during RNA-virus replication through aberrant polymerase copying events and have been detected in filovirus infection; their contribution to EBOV persistence is biologically plausible but should not be treated as an established universal mechanism [27,28]. At the population level, the unusually low sequence divergence of the 2021 Guinea outbreak lineage relative to viruses from the 2013–2016 epidemic is consistent with a prolonged period of markedly reduced replication before resurgence [6,29].

**Figure 3:**
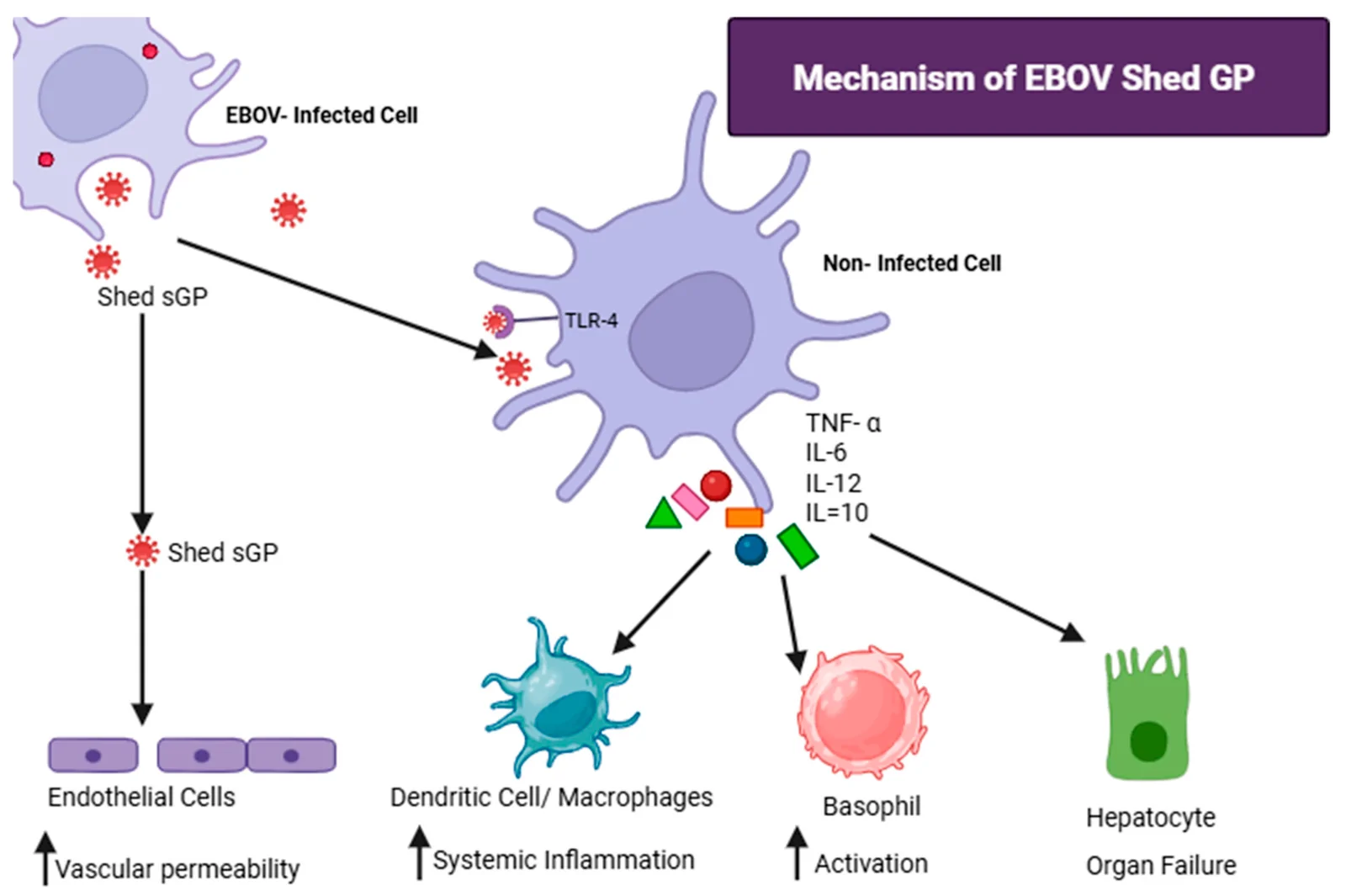
Effect of Ebola Shed GP on the Body. Arrows indicate the direction of the depicted effects, while upward arrows (↑) indicate increased activity or response. Created in BioRender. Ibrahim, M. (2026). https://BioRender.com/8xop0mx.

Two viral proteins have particular relevance to EBOV immune evasion. VP35 inhibits innate antiviral signaling at multiple levels, including interference with dsRNA sensing and downstream interferon induction [24,25]. VP24 inhibits interferon signaling by interfering with karyopherin-α-dependent nuclear transport of phosphorylated STAT1 [19,26]. Together, these proteins are well-established determinants of acute EBOV immune evasion. Their specific contribution to long-term persistence in reservoir cells remains less certain; recent human cerebral-organoid data instead support a more complex, cell-type-dependent interaction between persistent infection and local innate or inflammatory responses [30]. Figure 3 provides a schematic overview of selected systemic effects of EBOV-derived viral products and associated host responses.

EBOV primarily infects the mononuclear phagocyte system (monocytes, macrophages, dendritic cells), where viral amplification to high levels occurs and which, by producing pro-inflammatory cytokines, is a major contributor to systemic inflammation, followed by secondary infection of vascular endothelium and hepatocytes leading to increased vascular permeability, coagulopathy, and hepatic dysfunction in severe disease [25]. However, in the context of persistent infection, the following materially different tropism of retinal pigment epithelial cells in the eye, as well as of microglia and astrocytes in the CNS parenchyma and in the ventricular system, and of Sertoli cells, spermatogonia and other cells of the seminiferous epithelium in the testis, is of central relevance. This shift from immune and endothelial cells during acute systemic disease to tissue-resident, immunologically sequestered, largely non-immune cell populations during persistence recurs throughout this review and is central to understanding both why persistent infection has proven difficult to detect and model, and why it appears to coexist with cellular viability over extended periods in a way acute infection of myeloid and endothelial cells, with its pronounced cytopathic and pro-apoptotic consequences, does not [26,30].

During acute EVD, monocytes, macrophages, and dendritic cells are prominent early target cells, and infection is associated with systemic inflammatory responses and secondary organ involvement [31,32,33]. Persistent infection differs anatomically and biologically from this acute systemic phase. Evidence from survivor cases, non-human-primate studies, and human tissue models implicates macrophage-lineage cells in immune-privileged compartments, CNS-associated cells, retinal pigment epithelial cells, and cells within the male reproductive tract as potential reservoir-associated populations [34,35,36]. The relative contribution of each cell type differs by anatomical site and remains incompletely defined.

Acute EVD is characterized by rapid viral replication, dysregulated inflammatory signaling, lymphocyte bystander apoptosis, endothelial dysfunction, and coagulopathy [32,33,37]. Survivors, by definition, have controlled systemic viremia, yet a subset may retain viral RNA or replication-competent virus in anatomically sequestered compartments. This distinction between systemic clearance and compartmental persistence is central to the sections that follow.

## 4. The Anatomical and Physiological Basis of Immune Privilege Exploited by EBOV

### 4.1. The Central Nervous System and CSF-Ventricular System

The blood–brain barrier (BBB, tight-junctioned brain microvascular endothelial cells surrounded by end-feet of astrocytes and pericytes) and the structurally distinct blood–CSF barrier (tight-junctioned choroid plexus epithelial cells) are the paradigmatic blood–brain barrier (BBB); the absence of conventional lymphatics (slow drainage via glymphatics and meningeal lymphatics, which are less immunologically active); and the low expression of MHC class I/II on neurons, microglia and astrocytes under non-inflamed conditions. As a major reservoir cell type, microglia are CNS-resident myeloid cells derived from the yolk-sac that also contribute most effectively to local innate antiviral responses [38,39].

CNS persistence was the clinical anchor for the Scottish HWC with fatal-risk meningoencephalitis who had apparently recovered, having CSF with very high EBOV RNA and WGS showing direct descent from her original strain, which occurred about nine months after recovery [14]. NHP work at USAMRIID extended this single case into a system: NHP studies conducted by NHP work at USAMRIID showed that a subset of rhesus macaques survived experimental EBOV infection developed persistent, clinically silent infection in a few discrete areas (vitreous humor and adjacent ocular tissue, epididymal tubular lumina, and histiocytic brain inflammation) and was associated with CD68+ macrophage/monocyte-lineage cells, providing a unifying cellular theme of myeloid reservoirs within barrier protected tissue. A subsequent, mechanistically more detailed study (Science Translational Medicine) examined the interaction between standard-of-care monoclonal antibody (mAb) treatment and CNS persistence: macaques surviving lethal exposure following mAb treatment, in contrast to untreated survivors, harbored virus cleared from every other organ but persisting specifically within the brain ventricular system including choroid plexuses localized to infiltrating macrophages, accompanied by ventriculitis, choroid plexitis, and meningoencephalitis, and progressing to fatal recrudescent disease in roughly one in five treated survivors weeks to months after apparent recovery [35]. The investigators suggest that recruiting or altering circulating monocytes may also be able to cross a damaged or locally breached choroid plexus barrier like a “Trojan horse” to gain entry to the brain and may transport the virus a theory similar to the exosome model. These experimental findings demonstrate that CNS persistence and recrudescence can occur in antibody-treated non-human primate survivors. However, they do not establish that monoclonal antibody treatment causes reservoir formation or increases the risk of persistence in human survivors. Rather, they support the need for prospective studies evaluating whether acute-phase treatment influences subsequent reservoir establishment while accounting for relevant confounding factors [34].

### 4.2. The Ocular Compartment

The blood–ocular barrier limits access of plasma proteins, complement, and circulating leukocytes to the aqueous and vitreous compartments. It comprises the blood–aqueous and blood–retinal barriers and is complemented by local immunoregulatory mechanisms that restrain inflammation in order to preserve vision [12]. Aqueous-humor TGF-β2 is one component of this local regulatory environment, although evidence from non-Ebola ocular models should be used cautiously when inferring EBOV-specific mechanisms [40].

The eye provides direct evidence that an immune-privileged compartment can contain infectious, replication-competent EBOV after blood viremia has cleared. In a convalescent patient with severe panuveitis, viable EBOV was isolated from aqueous humor while blood RT-PCR was negative [13]. Cohort studies have subsequently documented substantial ocular morbidity among survivors, including uveitis and cataract, although ocular symptoms do not by themselves prove ongoing viral replication [10,41,42]. In rhesus macaque survivors, persistent EBOV has been identified in CD68-positive macrophage-lineage cells within ocular tissues [35]. Human retinal pigment epithelial cells are also susceptible to EBOV infection in vitro, supporting their consideration as a potential ocular reservoir cell type [36]. Complementary work with Sudan virus in non-human primate survivors has demonstrated persistence in the vitreous chamber and adjacent ocular tissue, predominantly in macrophages, indicating that ocular persistence is not unique to EBOV within the orthoebolaviruses [43].

### 4.3. Male Reproductive System

The testis is kept free from systemic immune surveillance by the blood–testis barrier (BTB), which is composed mainly of tight junctions that separate the seminiferous epithelium into a basal compartment for systemic immune surveillance and an adluminal compartment for developmental protection of meiotic and post-meiotic germ cells, which host potentially autoimmunogenic antigens [44]. In addition to this physical barrier, the testicular interstitium is also actively immunosuppressive: the interstitium contains an abundance of regulatory T cells (Tregs), local dendritic cells and macrophages are skewed tolerogenic, and interstitium contains Sertoli and Leydig cells that secrete the immunosuppressive molecule TGF-β and express the ligand for Fas constitutively [45]. In epidemiological terms, testicular/seminal persistence is the best-documented EBOV persistence phenotype because prolonged seminal RNA detection is supported by multiple longitudinal cohorts and selected sexual-transmission events are supported by genomic epidemiology. In Sierra Leone, Liberia, and Guinea, EBOV RNA remained detectable by RT-PCR in semen for many months after recovery, including reports extending beyond one year and, in isolated cases, beyond two years. These RT-PCR findings document persistence of viral RNA but should not be interpreted as evidence that replication-competent virus was present throughout the entire period of RNA detectability. Risk-factor analyses have associated prolonged seminal RNA detection with age, immunological characteristics including serum IgG3, and host genetic factors including *HLA*-C*03:04, although these associations do not by themselves establish causal mechanisms [46,47,48,49].

In crab-eating macaques, chronic infection of seminiferous tubules is primarily localized to Sertoli cells, and is associated with loss of spermatogenic cells, inflammatory infiltration and an actual disruption of inter-Sertoli tight-junction proteins (ZO-1, ZO-2), as well as infiltration of the tubules by immunosuppressive CD4+FoxP3+ Tregs, which suggests that MARV might actively exploit a pre-existing tolerogenic property of the testicular microenvironment [50]. Directly analogous findings (Sertoli tropism, seminiferous localization, associated tissue damage) have been reported for Sudan virus in Uganda NHP survivors [43]. For EBOV itself, in vitro data confirm human Sertoli cell susceptibility, and limited in vivo data localize EBOV genetic material to seminiferous tubules and testicular lumen, though the precise mechanism of BTB breach remains incompletely defined a priority research gap, as it is for MARV pathogenesis [35,46,47,48,49]. Notably, the flavivirus Zika virus (ZIKV) independently infects human Sertoli cells and disrupts barrier integrity via Axl receptor and local interferon modulation reinforcing that the testis is a broadly permissive niche across taxonomically unrelated viruses and suggesting Axl–interferon crosstalk as a potential therapeutic target worth exploring for EBOV [46].

### 4.4. Other Relevant Sites and Synthesis

The fetoplacental unit is itself immune-privileged by design (to tolerate the semi-allogeneic fetus), and while direct evidence of EBOV placental persistence is limited, at least one case documents EBOV RNA in the breast milk of a mother with no reported acute illness of her own interpreted as maternal persistence with lactational transmission to her breastfeeding infant and longer-term Guinean cohort follow-up (Postebogui, to 40 months) has similarly documented breast-milk RNA well beyond acute illness in a subset of survivors. In addition, a joint/periarticular site is a potential but hypothesised area, since chronic arthralgia in PES is common and the joint spaces are relatively immunologically silent [49,51].

**Figure 4:**
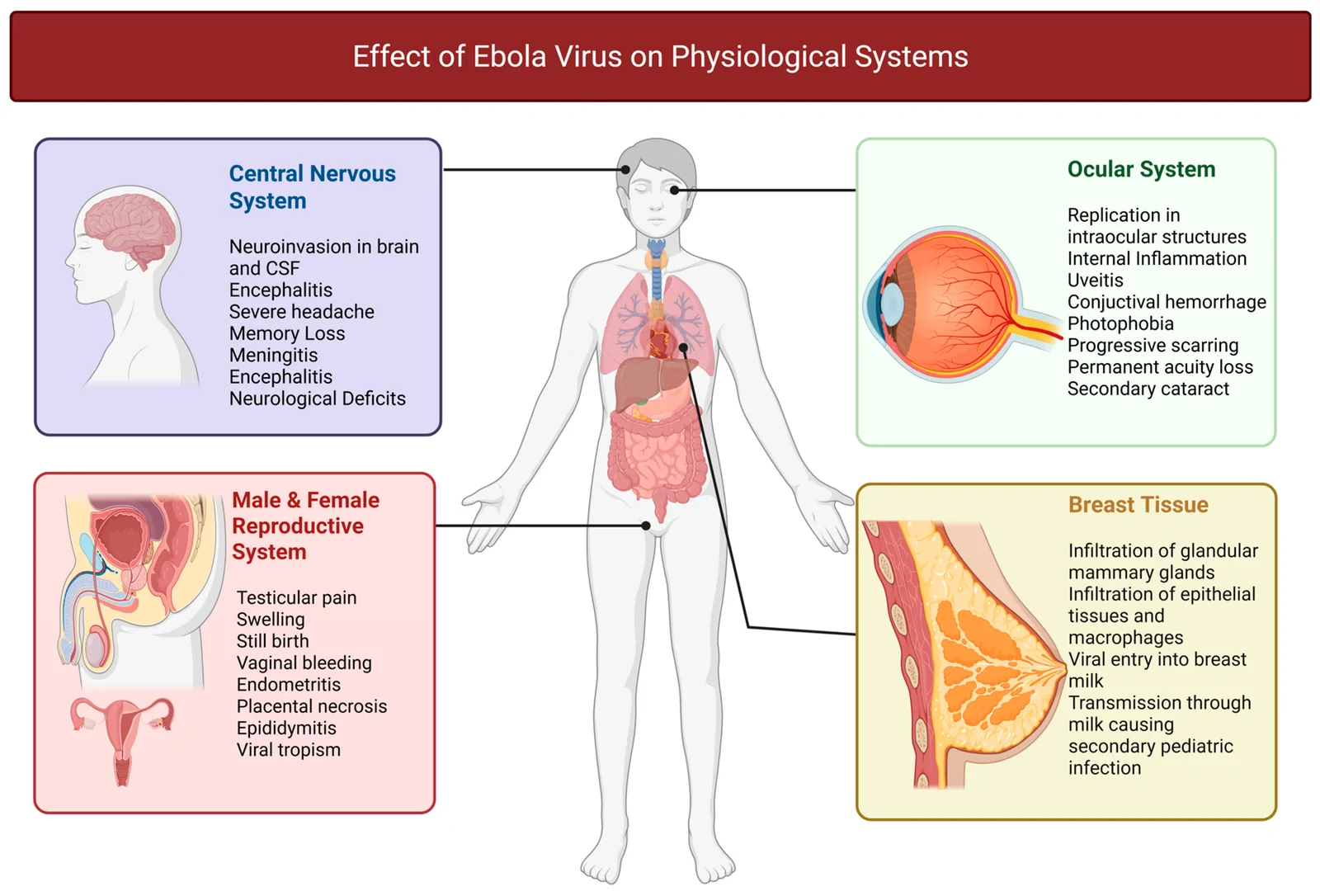
Effect of Ebola Virus on Systems. Created in BioRender. Ibrahim, M. (2026). https://BioRender.com/hzunsgj.

Across anatomical sites, the evidence does not support a single universal reservoir-cell lineage. Macrophage-lineage cells are prominent in experimental CNS and ocular persistence [34,35], whereas epithelial or specialized barrier-associated cells may contribute in other compartments, including retinal pigment epithelium and Sertoli-cell reservoirs described in related filovirus models [36,43,50]. The more consistent unifying feature is anatomical sequestration within tissue environments where barrier properties and local immune regulation can limit systemic immune access. The principal organ systems and anatomical compartments implicated in EBOV persistence are summarized in Figure 4. The characteristics and key findings of human cohort studies and case reports investigating EBOV persistence in semen, cerebrospinal fluid, and ocular fluid are summarized in Table 1.

**Table 1:** Characteristics of Human Cohort Studies and Case Reports on EBOV Persistence in Semen, CSF, and Ocular Fluid.

#	Study	Country/Outbreak	Design	Sample Size (N)	Specimen(s)	Detection Method	Key Quantitative Findings
1	Rodriguez et al. [52]	DRC (Kikwit, 1995)	Case series/early virological follow-up	Small (not specified)	Semen	RT-PCR	Earliest reported EBOV RNA detection in semen of a survivor at 82 days post-onset (RT-PCR); virus isolation positive to 82 days in one prior filovirus report cited by later studies
2	Sow et al. [53]	Guinea (Conakry, Macenta)	Cross-sectional	68 men; 98 semen samples	Semen	RT-PCR	8/68 men (11.8%) and 10/98 semen samples (10.2%) were RT-PCR positive; EBOV RNA was detected up to 9 months after disease onset, with declining positivity and RNA levels over time.
3	Deen et al. [46]	Sierra Leone	Prospective national cohort	220 enrolled; 210 provided an initial semen specimen	Semen	qRT-PCR (*NP* and *GP* gene targets)	57/210 men (27%) were RT-PCR positive on the initial specimen. Positivity declined with increasing time after discharge; in phase 2, positive specimens were obtained 4.1–15.7 months after discharge, with Ct ranges of 32.7–38.0 (NP) and 31.1–37.7 (GP).
4	Sissoko et al. [54]	Guinea (3 ETUs)	Prospective longitudinal cohort with murine infectivity sub-study	26 enrolled; 130 semen specimens	Semen	Quantitative real-time RT-PCR; infectivity tested via inoculation into immunodeficient mice (8 participants)	EBOV RNA detected in 86/130 specimens (from 19/26 participants, 73%); median duration of RNA detection 158 days post-onset (IQR 73–181; maximum 407 days at end of follow-up); median total follow-up 255 days (IQR 228–287) post-onset
5	Fischer et al. [47]	Liberia	Case report/short cohort	Small case-level report	Semen	RT-PCR	EBOV RNA detected in semen > 2 years (>730 days) after resolution of acute infection
6	Purpura et al. [55]	Liberia	Prospective national program (Men’s Health Screening Program)	429 initial cohort (program-wide enrollment larger)	Semen	RT-PCR	Longest documented RNA-positive interval: 565 days (~18.5 months) post-ETU discharge; program demonstrated the feasibility of serial semen RT-PCR testing with structured counseling.
7	Thorson et al. [56]	Sierra Leone	Prospective cohort (2 recruitment phases)	220 men (100 + 120 in two phases); mean age 31 years	Semen	Quantitative RT-PCR (qRT-PCR), biweekly follow-up until 2 consecutive negatives	Positivity 75.4% (95% CI 66.9–82.0%) at 180 days post-discharge; median persistence duration 204 days; positivity 26.8% (95% CI 20.0–34.2%) at 270 days; 6.0% (95% CI 3.1–10.2%) at 360 days; persistence at 1 year ~10.1% (95% CI 4.6–19.8%) among severe acute disease vs. ~0% among mild disease
8	Diallo et al. [5]	Guinea	Case report with genomic/epidemiological investigation	Single survivor; index case of 2016 transmission cluster	Semen	RT-PCR, whole-genome sequencing	Semen remained EBOV RNA-positive at 531 days after illness onset; epidemiological and genomic analyses supported sexual transmission approximately 470 days after onset and linked the transmitted lineage to the survivor.
9	Mate et al. [57]	Liberia	Case report with genomic investigation	Single transmission event (index survivor + new case)	Semen (survivor); blood (new case)	RT-PCR and whole-genome sequencing	Semen was EBOV RNA-positive 199 days after estimated illness onset. Genomic and epidemiological evidence supported sexual transmission, implying infective virus at least 179 days after onset; attempts to culture virus from the semen sample were unsuccessful.
10	Keita et al. [49]	Guinea (4 centers: Conakry, Forécariah, Macenta, Nzérékoré)	Prospective longitudinal Post-Ebogui cohort; follow-up to 40 months	802 survivors overall; semen samples from 277 men; multiple body-fluid sub-analyses	Semen and breast milk	RT-PCR (Ct cutoff ≤ 40.9 for positivity)	27/277 men (9.8%) had EBOV RNA detected in at least one semen sample. Estimated probability of remaining semen RNA-positive was 93.02% at 3 months, 60.12% at 6 months, 27.68% at 9 months, 10.32% at 12 months, 0.96% at 18 months, and 0.06% at 24 months; breast-milk RNA was also documented during long-term follow-up.
11	Dyal et al. [48]	Liberia	Case–case cohort (“early clearers” vs. “late clearers”)	131 male survivors	Semen (plus paired serology/genotyping)	qRT-PCR; HLA/KIR genotyping; IgG subclass ELISA	Late clearers significantly older (median 42.5 vs. younger; *p* < 0.001); fewer severe acute symptoms (*p* = 0.006); more lens opacifications (OR 3.9); higher total IgG3 (*p* = 0.005 at enrollment, *p* = 0.007 at ~936 days); *HLA*-C*03:04 associated with reduced odds of late clearance after adjustment for age (aOR 0.14, 95% CI 0.02–0.70)
12	Jacobs et al. [14]	United Kingdom (imported from Sierra Leone)	Single case report with genomic sequencing	1 patient	Cerebrospinal fluid (CSF)	RT-PCR (very high viral RNA burden reported); whole-genome sequencing	CSF EBOV RNA strongly positive ~9 months after apparent clinical recovery and prior negative blood PCR; sequencing confirmed direct descent from original 2014 infecting strain, excluding reinfection
13	Varkey et al. [13]	United States	Single case report	1 patient	Aqueous humor	Virus isolation (cell culture) plus RT-PCR; concurrent blood RT-PCR (negative)	Infectious, replication-competent virus isolated from aqueous humor ~2 months after apparent recovery, at a time when blood was PCR-negative
14	Sissoko et al. [51]	Guinea	Case report	1 mother–infant pair	Breast milk	RT-PCR	EBOV RNA detected in breast milk of a mother with no reported acute illness of her own; interpreted as likely source of mother-to-child transmission

## 5. Molecular Mechanisms Sustaining Viral Persistence

### 5.1. Defective Viral Genomes and Replication Cycling

Defective viral genomes (DVGs) are truncated or rearranged viral genome products generated by aberrant polymerase replication events. They can modulate replication and innate immune signaling in several RNA-virus systems, and DVGs have been identified in filovirus infection [27,28,58]. For EBOV, however, a DVG-based cycling model should be considered a proposed mechanism rather than a universally established explanation of persistence. Human cerebral-organoid work has demonstrated long-term productive EBOV persistence together with emergence of DVGs and genomic variants, providing direct evidence that these processes can coexist in a human, multicellular CNS-relevant model [30].

This proposed cycling model should not be treated as a universally sufficient explanation of EBOV persistence. Reviews of defective viral-genome biology emphasize that DVGs can influence antiviral immunity and persistence, but their effects are context dependent [59]. Accordingly, DVGs are best considered one candidate contributor within a broader persistence framework rather than the sole mechanism responsible for reservoir maintenance.

The most direct and mechanistically integrated evidence linking DVG biology to persistence in a human-relevant tissue context comes from a 2026 study using human iPSC-derived cerebral organoids three-dimensional neural tissue incorporating neurons, astrocytes, and, with continued culture, microglia developed collaboratively by investigators at the Icahn School of Medicine at Mount Sinai, the Bernhard Nocht Institute for Tropical Medicine, and the NIH Integrated Research Facility. EBOV established what investigators explicitly termed “productive persistence”: sustained, ongoing replication and infectious particle production, distinct from a fully quiescent latent state for 120 days in culture, with progressive infection of astrocytes and neurons, active recruitment and infection of migrating microglia, and the emergence of DVGs and defective particles alongside genomic subvariants bearing mutations not previously catalogued in human survivor sequence data, proposed (though not all functionally confirmed) to reduce replicative fitness [30]. This model is important because it reproduces several features relevant to CNS persistence in a multicellular human tissue context. Nevertheless, organoid observations remain preclinical and should not be interpreted as demonstrating that the same mechanisms operate quantitatively or uniformly in human survivors.

### 5.2. Cell-Type-Specific Interferon Attenuation and the Persistence Paradox

Investigators have demonstrated that the type I interferon (IFN-I) response to persistent infection of microglial and astrocyte cell lines (sustained infections up to 91 days) is qualitatively different than an overall tissue-wide response, which is mechanistically important as it suggests a more cell population specific permissiveness rather than the global interferon suppression that is currently assumed in most models of VP35/VP24 biology that are derived from myeloid and epithelial systems [26]. One surprising result is that infected organoids were able to produce detectable pro-inflammatory and IFN-I responses during the persistence phase but were not found to clear the infection; instead, inflammation actually increased over time, reaching its highest levels late in the 120-day window which investigators suggest could mirror the late onset, sometimes fatal meningoencephalitis seen in clinical settings. This “persistence paradox” redefines otherwise apparent immune quiescence as a dynamic, prolonged stalemate between partial control and partial clearance of the virus, and eventual “exacerbation” or “regression” into more overt tissue-inflammation as one possible immediate mechanism for clinical relapse delayed into late phase [30].

For conceptual clarity it is important to distinguish this phenotype from classical latency (as in herpesviruses or retroviruses) where the genome is kept transcriptionally silent, without continuous polymerase activity, until a precise reactivation stimulus. The organoid and NHP histopathology data instead support a model of low-level, ongoing, non-quiescent replication “productive persistence” denoting continued genome replication and, at least intermittently, infectious progeny production throughout, rather than an inert reservoir awaiting a discrete signal a distinction with direct therapeutic implication [60]. This distinction is important when considering therapeutic concepts borrowed from HIV research.

### 5.3. Cell-to-Cell and Exosome-Mediated Spread, and Genomic Evolution

Both the organoid study and complementary NHP analyses document that persistent-phase spread occurs substantially through direct cell-to-cell transmission across areas of close membrane apposition, in addition to conventional budding and extracellular release a well-recognized immune-evasion strategy across virus families (HIV, herpesviruses, hepatitis C) because it reduces exposure to extracellular neutralizing antibody [30]. Within already anatomically restricted privileged niches, cell-to-cell spread represents an additional, cell-biological layer of evasion operating atop the anatomical barrier itself. A conceptually related mechanism involves packaging of viral components into host-derived exosomes: Pleet and colleagues demonstrated that EBOV matrix protein VP40 is incorporated into exosomes from infected cells and that these VP40-containing exosomes cause dysfunction in uninfected bystander immune cells that take them up, extending the infection’s immunomodulatory reach beyond directly infected cells a viral “Trojan horse” strategy, since exosome uptake does not trigger the pattern-recognition alarm that direct viral exposure would, directly complementary to the monocyte-trafficking “Trojan horse” hypothesis for CNS ventricular seeding [61].

Polymerase proofreading is predicted to eventually lead to progressive divergence from the genome, which is what the organoid study directly observed: as replicative activity increased over time, mutations were observed that were likely to reduce replicative fitness, a likely adaptive trajectory for long-term tissue residency as a result of the dual potential for attenuation of replication to diminish cytopathic effects as well as to prevent re-activation of innate immune responses. At the population level, this same biology would suggest that the “low” divergence between the 2013–16 Guinea lineage and the 2021 lineage is consistent with a period of reduced replication (persistence) over many years, rather than suggesting continuous undetected transmission (which would result in a materially higher mutation burden) [6,29]. Evidence at the organoid level and population-genomic evidence are complementary, and this genomic signature is now becoming a valuable tool for retrospectively distinguishing the resurgence of infections from the reservoir from the emergence of new zoonotic spillover events or the presence of unrecognized community transmission [29].

## 6. Host–Virus Immunological Interactions in the Persistence Phase

### 6.1. Adaptive Humoral and Cellular Immunity

Most survivors develop virus-specific antibody responses to major ebolavirus antigens, and long-lived humoral responses have been documented after recovery from ebolavirus infection, including among survivors of Sudan ebolavirus disease [62]. Not all survivors are equally impacted: Of 787 survivors of Ebola from DRC (2018–2020), a quarter with serial antibody data (*n* = 358) were seronegative for at least two of three major antigens at discharge; antibody levels decreased significantly over time for all three major antigens, particularly anti-GP antibody, which is the primary neutralizing target and the antigen of all licensed monoclonal antibody therapeutics, and which continued to decline at a significant rate over months [63,64].

The cohort provides observational evidence relevant to the question of whether acute-phase therapeutic choice is associated with subsequent humoral immunity. Modeling of 36-month seropositivity probability showed differences among treatment groups, including lower estimated seropositivity after ansuvimab than after REGN-EB3, remdesivir, or ZMapp [63]. A possible explanation is that rapid antigen reduction after highly effective monoclonal-antibody therapy could alter the duration or magnitude of endogenous antigen exposure and thereby influence subsequent antibody kinetics; however, this mechanism has not been directly demonstrated. Separately, non-human-primate experiments have reported CNS persistence and recrudescence in antibody-treated survivors. These human observational data and NHP experimental findings address different biological endpoints and should not be interpreted as direct replication of the same effect. Importantly, these observations do not establish that monoclonal antibody therapy causes reservoir establishment. Baseline viral burden, acute-disease severity, timing of therapy, host immune characteristics, and survivor selection may influence both treatment-related outcomes and subsequent persistence. Accordingly, a treatment-dependent effect on reservoir establishment remains a testable hypothesis that requires prospectively designed human studies with appropriate control of confounding [34].

Much less is known about T-cell immunity during persistence, partly because of the logistical challenges in functionally characterizing T cells from resource-limited settings but also because persistence is a relatively new research question. The available evidence suggests that the immune system remains altered as far as past the resolution of acute illness, broadly consistent with the general rule, seen across other chronic viral diseases, that an immune system that needs to see the maximum response to acute illness may experience either exhaustion or dysregulation later on that may prevent a complete clearance of the virus at sequestered sites. On limited evidence, on a single case report, the possibility of a role of pre-existing immune dysregulation in determining persistence duration also exists but is not a finding; EBOV RNA has been found to persist in semen in a survivor with concurrent HIV infection.

### 6.2. Systemic–Local Immune Integration and the “Privilege Paradox”

Tissue-resident innate sentinels do not operate in isolation from systemic immunity. One proposed mechanism for seeding of the brain ventricular system involves trafficking of infected or virus-associated circulating monocytes across a regionally altered choroid plexus barrier; however, this causal sequence has not been fully demonstrated in human survivors [34]. Likewise, persistent or intermittent systemic inflammatory signals observed after EVD may reflect several processes, including post-infectious immune dysregulation or, in some individuals, antigenic stimulation from tissue-sequestered virus; these alternatives cannot currently be distinguished reliably. Immune privilege represents a coordinated set of host adaptations that limit potentially damaging inflammation in functionally sensitive tissues. We use the term ‘privilege paradox’ here as a conceptual framework rather than as an established EBOV persistence mechanism. The framework proposes that viral immune-evasion functions that evolved in the context of acute systemic infection could also facilitate survival after EBOV enters tissues in which host physiology already restricts immune surveillance. Available anatomical, experimental, and virological observations are compatible with this possibility, but they do not demonstrate that acute-phase immune-evasion mechanisms specifically pre-adapt EBOV for persistence in immune-privileged sites. Alternative explanations—including tissue-specific cellular permissiveness, stochastic reservoir seeding, local pharmacokinetics, differences in acute viral burden, and survivor-specific immune factors—may also contribute and remain incompletely resolved. Thus, the ‘privilege paradox’ is best regarded as a hypothesis-generating framework for integrating host anatomy and viral immune evasion, rather than as a proven causal model.

### 6.3. Host Genetic and Immunogenetic Determinants

Host immunogenetic factors may contribute to inter-individual differences in the duration of seminal EBOV RNA detection, but the available evidence remains associative rather than mechanistic. In a Liberian cohort comparing early and late clearers, HLA-C*03:04 and serum IgG3 were associated with differences in the duration of seminal EBOV RNA detection, alongside demographic and clinical variables [48]. A potential role for HLA-KIR interactions and NK-cell regulation has been proposed by analogy with other persistent viral infections; however, a specific causal *HLA-C*/KIR/NK-cell pathway has not been demonstrated experimentally or prospectively in EBOV survivors. Similarly, sustained IgG3 may reflect prolonged antigenic stimulation, but it cannot by itself identify the anatomical source of antigen or establish ongoing replication-competent infection. These observations should therefore be viewed as hypothesis-generating signals for future mechanistic and prospective studies rather than as established determinants that are currently suitable for clinical risk stratification.

Systemic host immunity may contribute to reservoir outcome alongside anatomical and viral factors, but the genetic evidence base remains limited. Candidate-factor studies, including the reported associations with HLA-C*03:04 and IgG3, are hypothesis-generating rather than causal [48]. To our knowledge, no adequately powered genome-wide association study has yet established host genetic determinants of EBOV persistence duration. Future studies should therefore prioritize independent replication and prospective integration of host genetics with virological endpoints.

## 7. Clinical and Public Health Consequences

### 7.1. Recrudescence and Post-Ebola Syndrome

Recrudescence refers to re-emergence of clinically apparent, virologically confirmed EVD in a previously recovered individual absent any plausible new exposure distinguished from reinfection and from relapse within a single, not-yet-resolved illness episode, and established with confidence chiefly through WGS demonstrating that the recurrent virus is phylogenetically nested within, and directly descended from, the individual’s own original lineage. The case that most closely meets these criteria is a clinically recovered British healthcare worker in December 2014, who was subsequently readmitted about 9 months later, with acute EBOV meningoencephalitis; CSF viral RNA level was extremely high, and the WGS confirmed that she was infected with the same strain the second time, ruling out reinfection. She eventually lived, with intensive supportive care, but the case has become the “hallmark” of CNS recrudescence, and fewer exhaustively documented cases of relapses were observed during later DRC outbreaks [14]. In an experimental rhesus macaque model, a subset of monoclonal-antibody-treated survivors developed brain-confined persistence and fatal recrudescent disease [33]. This model establishes that recrudescence can occur after treatment in non-human primates, but it should not be used to infer a quantified treatment-associated risk in humans.

**Figure 5:**
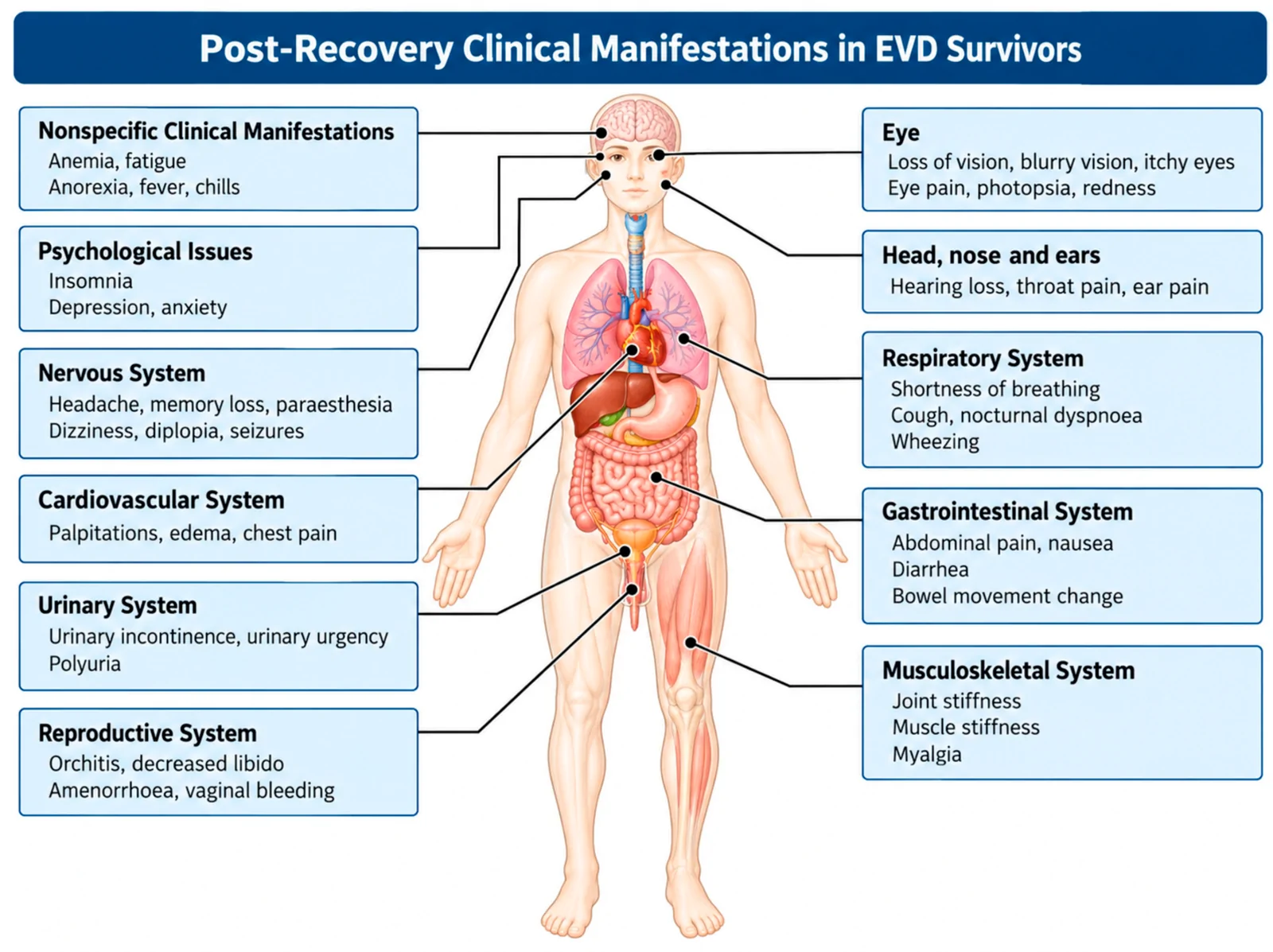
Post Ebola Manifestation in Survivors. Created in BioRender. Ibrahim, M. (2026). https://BioRender.com/kstk9yk.

More than three-quarters of survivors in the cohort study (750 survivors) had at least one PES sequela after 12 months, with musculoskeletal (49.7%) and neurologic (61.7%) sequelae most common and general/constitutional sequalae (38.4%) occurring in a significant proportion—observed across all acute-phase treatment groups, and in some cases with a detectable difference in proportions by treatment received (e.g., more neurologic sequelae in those who received REGN-EB3 than in those who received ZMapp or remdesivir, more musculoskeletal sequalae in those who received ZMapp than in those who received REGN-EB3 or remdesivir), that, while not yet mechanistically explained, adds a further dimension to the overarching theme that the choice of therapy during the acute phase may leave a detectable imprint in the long term. Many constitutional symptoms are clearly instances of a post-infectious immune-mediated process without evidence of ongoing virus replication and are more likely to improve within a year or two, thereby suggesting that PES is etiologically heterogeneous and includes these post-infectious manifestations [13,34]. Many others are for which there is direct virologic evidence that PES is linked to virus replication in tissue and are more likely to be linked to presentation of ongoing replication, most prominently uveitis (where infectious virus has been directly isolated from aqueous humor) and the most severe presentation, meningoencephalitis/relapse above, which is more likely to be linked to ongoing tissue-sequestered replication. However, there is a substantial gray zone between these extremes, and in the clinical literature, the most prominent example of which is chronic arthralgia, for which a plausible mechanistic case exists, but which has a dearth of direct virological confirmation from synovial fluid or periarticular tissue particularly because of the high prevalence of arthralgia and its functional burden. A useful methodologically useful clue is that all PES manifestations do not follow the same course; in some cohorts, constitutional and some musculoskeletal symptoms attenuate over one to two years, consistent with resolving post-infectious inflammation while ocular symptoms and some neurological symptoms persist or worsen a pattern that could be tested for in prioritizing further virological investigation of other PES manifestations [65]. The major post-Ebola manifestations described among survivors are summarized schematically in Figure 5.

### 7.2. Sexual and Mother-to-Child Transmission

Unlike CNS and ocular persistence, the major public-health consequence of seminal persistence is potential transmission to a sexual partner. Prolonged semen RT-PCR positivity alone does not establish infectiousness; stronger evidence requires epidemiological linkage and, ideally, genomic comparison of viruses from the survivor and recipient. The Liberia 2015 investigation identified unprotected sexual exposure to a male survivor approximately six months after his acute illness, and whole-genome sequencing provided molecular evidence consistent with sexual transmission [57]. A later Guinea investigation linked a 2016 transmission cluster to a survivor whose semen remained EBOV RNA-positive 531 days after illness onset; epidemiological and genomic analyses supported sexual transmission approximately 470 days after onset [5]. These events demonstrate that transmission can occur long after clinical recovery, but they should not be used to infer that all individuals with prolonged seminal RT-PCR positivity remain infectious for the full duration of RNA detectability.

In addition to these individual cases, national semen-testing programs documented prolonged EBOV RNA detectability; for example, the Liberian program reported RT-PCR-positive semen as late as 565 days after discharge. This observation defines duration of RNA detection, not duration of proven infectivity. Evidence concerning post-acute EBOV persistence in breast milk is considerably more limited than the evidence for seminal persistence. Longitudinal observations have documented EBOV RNA in breast milk after recovery, and a mother-infant investigation implicated breast milk as a plausible route of transmission despite the absence of reported acute maternal illness [49,51]. However, RT-PCR positivity alone cannot establish the presence of replication-competent virus, and the small number of documented cases prevents reliable estimation of either the duration or magnitude of transmission risk. Accordingly, mother-to-child transmission through breast milk should be regarded as a biologically plausible and clinically important concern supported by limited human evidence, rather than as a quantitatively defined reservoir phenomenon comparable to seminal persistence.

These individual- and transmission-level consequences converge, at the population level, in their most consequential documented manifestation: reignition of community outbreaks. The paradigmatic case is the February–June 2021 outbreak near Gouéké, Nzérékoré region, Guinea epicenter of the 2013–2016 epidemic. WGS of virus from twelve patients showed the responsible lineage nested within, rather than separate from, the 2013–2016 clade, with divergence substantially lower than expected under continuous unrecognized transmission most parsimoniously explained by multi-year reduced replicative activity (persistence) followed by reactivation and renewed transmission. The outbreak produced 16 confirmed and 7 probable Guinean cases (12 deaths), alongside a linked DRC cluster of 11 confirmed and 1 probable case (6 deaths). The case in Butembo, DRC, was confirmed in February 2021 and genomically connected to the 2018–2020 Nord Kivu/Ituri epidemic (which ended in June 2020), further indicating the possibility of reservoir reactivation or transmission from survivors to contacts, instead of new introduction; the few cases since then that have been genomically connected to that same epidemic lineage suggest that these cases are not an isolated event, but are an ongoing, recurring trend [6].

## 8. Diagnostic and Surveillance Considerations

### 8.1. RNA Detection Versus Infectivity: The Central Methodological Problem

Many quantitative persistence estimates—including prolonged seminal and breast-milk positivity—derive from RT-PCR detection of viral RNA, a highly sensitive method that cannot, by itself, distinguish intact replication-competent virions from non-infectious RNA fragments, defective genomes, or residual nucleic acid. This distinction is fundamental: an RT-PCR-positive specimen is not necessarily infectious, and an RT-PCR-negative specimen does not by itself prove sterilizing eradication of a previously infected tissue compartment. Accordingly, durations reported from RT-PCR-based cohorts throughout this review refer to duration of RNA detectability unless recovery of replication-competent virus or documented transmission is explicitly stated. Virus isolation, genomic linkage, longitudinal clinical relapse, and tissue-based evidence therefore represent distinct and generally stronger evidentiary endpoints than RT-PCR positivity alone.

The few available linkages that are most closely related to the present situation are those between the cycle threshold (Ct) value detected by RT-PCR and the possibility of virus recovery: for blood from acute patients, successful virus isolation was not obtained from specimens with a Ct value above 35.5 for the *NP* gene target; for semen from US-tested survivors, the highest Ct for which a viable virus was isolated was 30, while the isolates from twelve specimens with higher Ct values were uniformly negative [57,66]. This is a working principle lower Ct, higher probability of infectivity that is used to aid clinical counselling, although it is far from a valid generalizable correlate of infectivity the Ct–infectivity relationship is a different one for each specimen type, platform, and presumably for tissue-specific factors: Seminal plasma composition, local antibody levels, proportion of defective- versus full-length genomes [15]. In the first documented case of sexual transmission where the virus was detected by genome sequencing (Liberia in 2015), RNA was detected in the survivor’s semen, but no infectious virus was isolated from the specimen, likely due to specimen handling or timing or to technical limitations, highlighting additional practical limitations of isolation: it is not as sensitive as RT-PCR; it requires BSL-4 cell-culture capability; it is technically demanding; and it is impractical for routine surveillance of thousands of enrolled survivors. The overall result is an ongoing incompleteness in the test, namely that while RT-PCR is scalable, it cannot confirm infectivity, and isolation can confirm infectivity, but is far too insensitive and resource hungry to be implemented routinely leaving in most real-world scenarios, the clinician and survivors to make decisions about risk reduction based on RNA positivity alone [46,57,67].

### 8.2. Surveillance Programs and Site-Specific Diagnostics

The public-health response to seminal persistence unfolded in an iterative manner, in the face of growing evidence. Based on the very few reports of virus-isolation that had come before the epidemic, the pre-epidemic guidance was for 90 days of abstinence or condom use, when there was growing evidence that the virus persisted for much longer. WHO’s response directly to the ongoing transmission investigation in Liberia, in May 2015, was to revise their guidance to include serial semen (RT-PCR) testing of all male survivors until two consecutive negative results and to provide condoms and structured counselling at treatment-unit discharge, further revised in January 2016 as more evidence emerged a system that appeared to be continuously adjusting guidance in near real time. Liberia’s Men’s Health Screening Program, launched in July 2015, is the most fully documented; it has enrolled male survivors (15+ y.o.) for serial monitoring and counselling based on the current guidelines from the WHO; Sierra Leone Ebola Virus Persistence Study created a structured, replicable risk-reduction counselling model for translating the quantitative test results into actionable counselling. Persistent gaps remain in program scope, particularly extension to ocular fluid and breast-milk testing [55,68].

There can be significant variation in diagnostic considerations between sites. Sampling of CSF is reserved for suspected cases of CNS reservoir disease (invasive lumbar puncture) and is usually only done in symptomatic individuals, where the risk-benefit ratio in asymptomatic individuals is not in favor. Neuroimaging may be used if available. The invasiveness concerns are similar with ocular fluid sampling (aqueous or vitreous) which is only performed when uveitis is symptomatic and not as routine screening. Semen testing procedures, on the other hand, are relatively non-invasive and underlie the above national programmes, but are subject to Ct-infectivity limitations as discussed [69]. Single-cell and spatial transcriptomics provides tools to directly characterize reservoir cell populations and their local immune microenvironment at unprecedented resolution; use of genomic surveillance, which has proven effective during the resurgence investigations, will provide a population-level diagnostic tool to distinguish reactivation of reservoir cells from new spillover; continued efforts to develop a validated and generalizable correlate of infectivity perhaps by leveraging defective-versus-full-length genome discrimination, rather than relying on Ct value alone represents the field’s most important unmet diagnostic need [13,14].

## 9. Therapeutic Strategies and Interventions

The therapeutic approaches discussed below differ substantially in their level of supporting evidence. Remdesivir is the only intervention discussed here with randomized clinical-trial data specifically addressing persistent seminal EBOV RNA. Monoclonal antibodies and vaccines have established roles in acute-disease treatment or prevention, but their effects on established persistent reservoirs remain uncertain. By contrast, strategies involving manipulation of defective viral genomes, exosome biogenesis, reservoir-directed immunomodulation, or ‘shock-and-kill’-like approaches are conceptual or preclinical proposals and have not been validated as therapies for EBOV persistence. These categories should therefore not be interpreted as representing equivalent stages of therapeutic development.

### 9.1. Direct-Acting Antivirals

Remdesivir (GS-5734), a nucleotide-analogue prodrug targeting the viral RNA-dependent RNA polymerase, has the most direct randomized clinical-trial evidence relevant to persistent seminal EBOV RNA. Preclinical pharmacokinetic and efficacy studies in non-human primates provided a rationale for evaluating the drug in survivors [70]. PREVAIL IV (NCT02818582) was a randomized, double-blind, placebo-controlled phase 2 trial in Liberia and Guinea in which 38 male survivors with documented persistent seminal EBOV RNA received five days of intravenous remdesivir or placebo. The principal finding was a higher mean semen RT-PCR assay-negativity rate during months 2–6 after treatment in the remdesivir group than in the placebo group [71]. This result provides randomized evidence that remdesivir can reduce the detectability of EBOV RNA in semen. It does not, however, demonstrate eradication of an anatomical reservoir, elimination of replication-competent virus, or prevention of subsequent sexual transmission. The small sample size, reliance on RT-PCR endpoints, and absence of direct tissue-reservoir or infectivity endpoints limit broader interpretation, and confirmatory studies are required. No analogous randomized clinical evidence currently establishes efficacy against CNS or ocular persistence.

### 9.2. Monoclonal Antibodies and Vaccination

The monoclonal antibody therapeutics Inmazeb (REGN-EB3; atoltivimab, maftivimab, and odesivimab) and Ebanga (ansuvimab) substantially improved survival in acute EVD and are established acute-phase therapies based on the PALM trial [72]. Their effects on the establishment, maintenance, or clearance of persistent tissue reservoirs in human survivors remain uncertain. Observational differences in subsequent endogenous antibody kinetics and experimental observations of CNS persistence in antibody-treated non-human primates generate a hypothesis that acute-phase treatment could influence post-acute reservoir biology, but these data do not establish causality. Potential confounding by baseline viral burden, disease severity, timing of therapy, host immune characteristics, and survivor selection must be considered. These observations provide a rationale for prospectively evaluating reservoir outcomes and, where appropriate, sequential reservoir-directed antiviral strategies; they do not currently support changing established acute-phase monoclonal antibody treatment recommendations.

The rVSV-ZEBOV vaccine (Ervebo) has established efficacy for prevention of EVD. In the Ebola Ça Suffit! ring-vaccination trial, the randomized comparison estimated vaccine efficacy at 100% (95% CI 68.9–100), while the broader analysis including all eligible rings yielded an estimate of 100% (95% CI 79.3–100) [73]. These findings support prevention in exposed populations but do not establish a therapeutic effect on persistent reservoirs in survivors. Evidence is currently insufficient to determine whether vaccination or revaccination of survivors can clear established EBOV from immune-privileged sites. Any reservoir-directed vaccination strategy should therefore be considered investigational rather than an evidence-based clinical recommendation.

### 9.3. Conceptual and Preclinical Reservoir-Directed Approaches

The approaches in this subsection are hypothesis-generating proposals rather than validated EBOV persistence therapies. Manipulation of the full-length/defective-genome ratio has been proposed conceptually because DVGs may alter viral replication dynamics, but no clinical evidence currently demonstrates that deliberately shifting this ratio is safe or effective for clearing EBOV reservoirs. Similarly, analogy with the HIV ‘shock-and-kill’ paradigm is conceptual and should not be interpreted as evidence that latency-reversal strategies are applicable to EBOV, whose documented CNS-relevant persistence is productive rather than classical proviral latency [30,74]. VP40-containing extracellular vesicles provide a mechanistic rationale for studying vesicle biology, but pharmacological inhibition of exosome biogenesis has not been validated as a reservoir-directed EBOV treatment [61]. Intrathecal, intraventricular, or intravitreal drug delivery may merit future investigation when systemic tissue penetration is inadequate, but these routes have not been clinically evaluated for persistent EBOV. Host-directed immunomodulation is even more uncertain because increasing local immune activity in immune-privileged tissues could itself cause tissue-damaging inflammation. These concepts should therefore be prioritized for mechanistic and preclinical evaluation before clinical translation.

When taken in aggregate, the therapeutic evidence remains uneven. Randomized clinical-trial evidence exists for remdesivir reducing seminal EBOV RNA detectability, whereas no comparable randomized evidence establishes clearance of CNS or ocular reservoirs [70,71]. Monoclonal antibodies are established life-saving acute-phase therapies [72], but their relationship to subsequent reservoir establishment in humans remains unresolved despite relevant NHP observations [34]. Other reservoir-directed strategies remain conceptual or preclinical and should not be presented as equivalent to clinically validated interventions. The key findings from interventional clinical trials and public health studies relevant to EBOV persistence management are summarized in Table 2.

**Table 2:** Interventional Clinical Trials and Programmatic (Public Health) Studies Relevant to EBOV Persistence Management.

#	Study/Program	Country/Setting	Design	Population (N)	Intervention	Comparator	Primary Outcome(s) and Results	Reference
1	PREVAIL IV (GS-5734/remdesivir semen trial)	Liberia, Guinea	Randomized, double-blind, placebo-controlled, phase 2	38 male survivors with documented persistent seminal EBOV RNA (≥1 of ≥2 samples positive within 42 days of enrollment)	Intravenous remdesivir, 100 mg daily × 5 consecutive days	Matching placebo	Significantly increased semen RT-PCR negativity (assay negativity rate) over 2–6 months post-treatment vs. placebo; safety profile acceptable; investigators noted need for larger confirmatory trial	[71]
2	PALM (Pamoja Tulinde Maisha) trial	DRC (2018–2020 Nord Kivu/Ituri epidemic)	Randomized controlled trial, 4 arms	Acute EVD patients (multi-hundred enrollment across 4 arms)	REGN-EB3, ansuvimab (mAb114), ZMapp	Remdesivir (and cross-arm comparisons)	Overall mortality: REGN-EB3 33.5% vs. ZMapp 51.3%; viral-load-stratified: high viral load (Ct < 22) 63.6% (REGN-EB3) vs. 86.2% (ZMapp); low viral load (Ct ≥ 22) 11.2% (REGN-EB3) vs. 25.8% (ZMapp); trial stopped early for efficacy in the REGN-EB3/ansuvimab arms	[72]
3	Les Vainqueurs d’Ebola antibody-kinetics cohort	DRC (2018–2020 epidemic survivors)	Prospective observational cohort	787 enrolled; 358 with serial antibody data	(Observational: comparison by acute-phase treatment received)	Cross-treatment-group comparison (ansuvimab, REGN-EB3, remdesivir, ZMapp)	24% seronegative for ≥2 of 3 major antigens (GP, NP, VP40) at ETC discharge; continuous antibody decline across all groups; 36-month seropositivity probability: ~53.6% (ansuvimab) vs. ~73.5% (REGN-EB3) vs. ~76.8% (remdesivir) vs. ~78.5% (ZMapp)	[64]
4	Ebola Ça Suffitring vaccination trial	Guinea (final phase of West African epidemic)	Cluster-randomized (ring vaccination), open-label	Contacts and contacts-of-contacts of confirmed cases (immediate vs. delayed vaccination clusters)	rVSV-ZEBOV (single dose)	Delayed vaccination (21-day deferral)	Randomized comparison: vaccine efficacy 100% (95% CI 68.9–100); broader analysis including all eligible rings: 100% (95% CI 79.3–100). No cases occurred ≥10 days after vaccination in the immediate-vaccination clusters.	[73]
5	Liberia Men’s Health Screening Program (MHSP)	Liberia	Prospective national public-health program (established July 2015)	429 (initial cohort); program-wide enrollment larger	Serial RT-PCR semen testing + structured behavioral counseling, condom provision	Pre-program (90-day abstinence guidance) as historical comparator	Longest documented positive interval: 565 days post-discharge; program implemented serial semen testing, structured counseling, and condom provision and informed evolving survivor-care guidance.	[55]
6	Sierra Leone Ebola Virus Persistence Study (EVPS) behavioral counseling protocol	Sierra Leone	Program development/implementation study	Enrolled survivor cohort (linked to Deen et al. [46] virological cohort)	Structured, individualized pre-test and post-test risk-reduction behavioral counseling calibrated to qRT-PCR/isolation results	N/A (protocol development/implementation study)	Established replicable, test-result-calibrated counseling framework subsequently adapted by other regional testing programs	[75]

## 10. Comparative Perspective: EBOV Persistence Among Other Viruses

Placing EBOV persistence in the larger context of viral persistence in privileged tissues, a much more established research field in general, provides helpful parallels and enlightening contrasts. HIV-1 is a paradigm case: CNS persistence is tightly connected to the blood–brain and blood–CSF barriers and perivascular macrophages/microglia are the major reservoir, even in the presence of long-term suppressive antiretroviral therapy [76,77]. Recent synthesis suggests that the CNS is not a single sanctuary but rather a “sanctuary within a sanctuary”, with important inter-regional variation in drug penetration, and cellular composition which is striking with respect to EBOV being tightly bound to the ventricular system and choroid plexus in particular and not diffusely distributed throughout parenchyma. Direct cross-viral precedent for local-inflammation-driven reservoir destabilization as a plausible EBOV recrudescence trigger a particularly instructive clinical precedent: at least one reported case demonstrates that an unrelated CNS inflammatory insult (JC virus-associated inflammation) in an ART-suppressed patient results in a substantial, corticosteroid-responsive increase in CSF HIV-1 viral load, which is then restored to suppression by anti-inflammatory treatment alone [77]. The same kind of blood–testis barrier architecture underlies HIV-1 persistence, and direct evidence of compartmentalized, independently evolving viral populations in men is provided by documented discordant drug-resistance mutation profiles in blood and semen, which are comparable to the Guinean survivor’s distinct VP30 mutation [77,78].

Zika virus (ZIKV) is the only virus in the field with a mechanistically detailed model of persistence in human Sertoli cells, which replicate ZIKV to high levels for up to 6 weeks in culture, which is dependent on the Axl receptor tyrosine kinase; infection results in extensive transcriptional reprogramming, the upregulation of FGF2, which is itself capable of enhancing replication—a self-reinforcing autocrine loop, rather than a mere byproduct of reduced surveillance [79,80]. ZIKV infection also leads to the induction of the RNA binding protein tristetraprolin, which inhibits local type I/III interferon another pathway that results in an equivalent functional effect, independently highlighting the need for local interferon attenuation for persistent infection. Of note, ZIKV sexual transmission is documented in at least thirteen countries and has been found to persist in the semen for up to 370 days, which is of the same order of magnitude as EBOV, but less than the maximal persistence of 531 days seen with EBOV, highlighting that the persistence in the testicles is a broadly conserved phenomenon across taxonomically unrelated viral families. A methodological caveat: not every human testicular cell line is equally able to support persistence of ZIKV (and all organoid and cell-line persistence data, including EBOV data, should be viewed in this light) [80,81].

Historically, the best examples of defective genome, CNS persistence are the measles virus and subacute sclerosing panencephalitis (SSPE) which was discovered decades prior to considering the potential for EBOV to persist in the CNS. The accumulation of clustered mutations in the matrix (M) protein, the functional equivalent of EBOV VP40, that is known to reduce the amount of infectious particles released from cells, and increase the extent of cell-to-cell spread due to increased fusion, is a hallmark that maps exactly onto the EBOV phenotype, that is, reduced infectious particle output and increased cell-to-cell spread (analogue to DVG-mediated attenuation) [80]. Measles virus also generates defective interfering genomes frequently and early, particularly under impaired viral C-protein function, and suppressed polymerase activity is understood as a necessary condition for transformation into the persistently infecting SSPE variant direct, mechanistically confirmed precedent, established well before the analogous EBOV organoid findings, for the general principle that attenuated (not absent) polymerase activity permits stable CNS persistence [82]. A significant caveat limits full cross-viral extrapolation: SSPE develops overwhelmingly following early-childhood measles infection, with its extended latency proposed to relate partly to host immunological immaturity at primary infection a host-age-dependent factor with no established EBOV analogue, since EBOV persistence is documented predominantly in adult survivors with no evidence linking age at acute infection to subsequent persistence risk [83,84].

Considered together, EBOV persistence is best understood as one instance a clinically and epidemiologically consequential one, given its outbreak-reignition potential of a broader, convergently evolved viral strategy for exploiting privileged tissue that recurs across taxonomically unrelated virus families. Common principles that are shared by HIV-1, ZIKV, measles and EBOV, and that recur across these pathogens, are: preferential use of myeloid or myeloid-like reservoir cells, local (cell intrinsic) interferon attenuation through multiple molecular pathways that all converge to the same functional outcome, a defect in the genome that is sufficient to achieve a stable, low-cytopathic persistent state, and a strong dependence on cell-to-cell spread to minimize extracellular antibody exposure. [5,6,13,14,30,34,35]. Cross-viral comparisons should therefore be used to generate testable hypotheses, not to imply that mechanisms established in other persistent infections have already been demonstrated for EBOV. A comparison of the principal reservoir sites, cellular tropism, and proposed persistence mechanisms of EBOV and other viruses is presented in Table 3.

**Table 3:** Comparison of Ebola to other viruses.

Pathogen	Primary Sanctuary Reservoir(s)	Primary Tropism	Main Mechanism of Attenuation/Persistence	Reference
HIV-1	CNS, Lymph nodes, GALT	Microglia, Macrophages, CD4+ T cells	Integrated latent provirus; low transcriptional activity; cell-to-cell spread across virological synapses.	[85]
ZIKV	Testes (Sertoli cells), CNS, Eye	Sertoli cells, Neural progenitor cells, Macrophages	Sertoli-cell permissiveness; AXL-facilitated entry/immunomodulation in human Sertoli models; blood–testis barrier disruption demonstrated in mouse studies.	[79,80]
Measles (SSPE)	CNS (Neurons, Glia)	Neurons, Astrocytes	Matrix (M) protein mutations preventing budding; loss of C protein generating DVGs; extensive cell-to-cell fusion.	[82]
EBOV	CNS, eye, male reproductive tract	Macrophage-lineage cells; neural/glial cell types in organoid models; ocular epithelial cells; male-reproductive-tract cells	Productive persistence with site-specific cellular reservoirs; cell-to-cell spread and DVG/genomic variation demonstrated in human cerebral organoids.	[13,30,34,35,36]

## 11. Knowledge Gaps and Priority Research Directions

The methodological basis of the field the lack of a clear relationship between RNA detection and true infectivity makes the interpretation of almost every quantitative claim in this manuscript uncertain; the specific development of an informative, scalable correlate of infectivity (possibly based on defective versus full-length genome discrimination rather than based on Ct value alone) is therefore the single most important methodological recommendation in the present manuscript. Mechanistically, the trigger(s) for reservoir reactivation remain unknown: neither the proposed KIR-NK cell pathway nor the general principle, drawn from HIV-1 precedent, that intercurrent inflammatory illness may destabilize an otherwise quiescent reservoir, has been directly, prospectively tested in EBOV-infected or -exposed individuals a hypothesis-driven investigation of intercurrent illness as a reactivation trigger is a well-motivated near-term priority. The host genetic and immunogenetic evidence base is still limited and underpowered when compared to the precedent set by HIV-1, and to date, no genome-wide association study of persistence duration has been reported, although the already-biobanked survivor cohort specimens are ideal for such analysis without additional recruitment, representing a low-cost, high-yield opportunity. A validated immunological correlate of protection against relapse is lacking, unlike the acute-phase vaccine-efficacy correlates literature, and would directly influence risk-stratified counselling, as well as serve as an objective endpoint to assess future immunomodulatory or therapeutic-vaccination strategies.

Whether acute-phase therapeutic choice influences subsequent reservoir establishment remains unresolved. The observations discussed in this review, including treatment-associated differences in endogenous antibody kinetics and experimental findings in non-human primates, justify prospective investigation but do not establish a treatment-specific causal effect in human survivors. Future prospective cohorts should therefore evaluate reservoir-related outcomes across acute-phase treatment groups while accounting for baseline viral burden, disease severity, timing of therapy, host factors, and other potential confounders. More broadly, no adequately powered human study has established whether any current acute-phase therapy increases or decreases the probability of long-term tissue persistence. No synovial-fluid or periarticular-tissue study has directly resolved whether chronic post-Ebola arthralgia reflects persistent virus in joint-associated tissues; appropriately designed studies could help distinguish persistent infection from post-infectious inflammatory mechanisms.

The persistence research agenda also has important survivor-centered social and ethical implications. Long-term follow-up, repeated testing, and renewed concern about transmission can impose psychosocial burdens and may reinforce fear or stigma toward survivors, particularly when a positive RT-PCR result is incorrectly interpreted as proof of infectiousness. Longitudinal data from Liberian survivors have documented persistent Ebola-related stigma after recovery, including fluctuations associated with periods of disease re-emergence [86]. Reservoir surveillance should therefore be accompanied by careful risk communication, confidentiality safeguards, psychosocial support, and counseling that clearly distinguishes RNA detection from demonstrated infectiousness. These measures are important both ethically and operationally because stigmatizing surveillance may undermine survivor engagement with long-term follow-up programs.

Several of these gaps are not unrelated to true ethical and logistical limitations: longitudinal invasive CSF, ocular or testicular sampling in asymptomatic survivors does raise a risk-benefit calculus that is hard to reconcile with conventional research-ethics frameworks even if the knowledge derived from sampling has clear group-level value, but in favor of minimally invasive sampling of blood, semen, tears, or saliva and statistical inference of tissue-level dynamics wherever possible. Sustaining the survivor cohort infrastructure, genomic surveillance capacity, and BSL-4 laboratory capability this research agenda requires demands long-term, dedicated funding commitments that have historically proven vulnerable to de-prioritization once an epidemic’s acute phase concludes and public attention moves elsewhere a structural funding challenge this review identifies as, in practice, as significant a barrier as any purely technical limitation.

## 12. Conclusions

This review shows that Ebola virus disease cannot be viewed solely as an acute infection ending with death or apparent systemic recovery. Evidence supports persistence of EBOV or EBOV RNA in immune-privileged sites, particularly the central nervous system, eye, and male reproductive tract, but the strength of evidence differs substantially among anatomical sites and endpoints. RT-PCR detection of viral RNA, recovery of replication-competent virus, and documented transmission should therefore remain explicitly distinguished. Restricted immune surveillance and tissue-specific host environments provide a plausible anatomical basis for persistence, whereas several proposed molecular and immunological mechanisms—including treatment-dependent reservoir establishment—remain incompletely established and require prospective testing. Vaccines and monoclonal antibodies have transformed prevention and acute-phase treatment, while the ability of currently available interventions to eliminate established tissue reservoirs remains uncertain. Future research should prioritize validated correlates of infectivity, determinants of reservoir establishment and relapse, appropriately controlled longitudinal studies of treatment-related outcomes, and reservoir-directed interventions supported by evidence of tissue penetration and biological activity. Such work is important for survivor care, risk communication, and prevention of persistence-associated transmission or outbreak resurgence.

## Data Availability

No new data were created or analyzed in this study. Data sharing is not applicable to this article.
